# Demonstration of proof-of-concept of *StrokeShield* system for complete closure and occlusion of the left atrial appendage for non-valvular atrial fibrillation therapy

**DOI:** 10.1371/journal.pone.0253299

**Published:** 2021-06-22

**Authors:** Mark S. Slaughter, Gretel Monreal, Steven C. Koenig, Guruprasad A. Giridharan, Landon H. Tompkins, Jorge H. Jimenez

**Affiliations:** 1 Department of Cardiovascular and Thoracic Surgery, University of Louisville, Louisville, Kentucky, United States of America; 2 Department of Bioengineering, University of Louisville, Louisville, Kentucky, United States of America; 3 Cor Habere Corporation, Louisville, Kentucky, United States of America; Bay Area Cardiology and Vascular/HCA West - USF Brandon Regional Hospital Cardiovascular Disease Fellowship, UNITED STATES

## Abstract

In the US, the most significant morbidity and mortality associated with non-valvular atrial fibrillation (NVAF) is embolic stroke, with 90% of thrombus originating from the left atrial appendage (LAA). Anticoagulation is the preferred treatment for the prevention of stroke in NVAF patients, but clinical studies have demonstrated high levels of non-compliance and increased risk of bleeding or ineligibility for anticoagulation therapy, especially in the elderly population where the incidence of NVAF is highest. Alternatively, stroke may be preventing using clinically approved surgical and catheter-based devices to exclude or occlude the LAA, but these devices continue to be plagued by peri-device leaks and thrombus formation because of residual volume. To overcome these limitations, Cor Habere (Louisville, KY) and the University of Louisville are developing a LAA closure device (*StrokeShield*) that completely occludes and collapses the LAA to minimize the risk of stroke. The *StrokeShield* device is a collapsible occluder (nitinol reinforced membrane) that completely covers the LAA orifice with an expandable conical coil anchor that attaches to the myocardium. The device is designed for catheter-based delivery and expands to completely occlude the LAA orifice and collapse the LAA. The primary advantages of the *StrokeShield* system are a completely sealed LAA (no peri-device flow or residual space) and smooth endothelialized connection to the left atrial wall with minimal risk of cardiac bleeding and tamponade. We tested proof-of-concept of a prototype *StrokeShield* device in acute (n = 2) and chronic 60-day (n = 2) healthy canine models. Acute results demonstrated that the conical coil securely attached to the myocardium (5N pull-out force) and the Nitinol umbrella fully deployed and covered the LAA ostium. Results from the chronic implants demonstrated long-term feasibility of device placement with no procedural or device-related intra- or post-operative complications, secure placement and correct positioning of the device with no device migration. The device successfully occluded the LAA ostium and collapsed the LAA with no interference with the mitral valve, circumflex coronary artery, or pulmonary veins. Necropsy demonstrated no gross signs of thrombus or end-organ damage and the device was encapsulated in the LAA. Histology demonstrated mature neointima covering the device with expected foreign body inflammatory response. These early positive results will help to guide the iterative design process for the continued development of the *StrokeShield* system.

## Introduction

The incidence of nonvalvular atrial fibrillation (NVAF) is increasing worldwide and represents a major health care burden [1, 2]. Despite advances in medical care, prognosis for NVAF remains poor due to the risk of embolic stroke from flow stagnation, especially in the left atrial appendage (LAA). In the US, there are 2.6 to 6.1 million patients with NVAF, which is expected to increase to 12 million by 2050 [3, 4]. The treatment of NVAF is estimated to cost between $16 and $26 billion annually [3, 5], with AF-related hospitalizations accounting for over half of these costs (52%) [5]. The most significant morbidity and mortality associated with NVAF is embolic stroke, with 90% of thrombus originating in the LAA [6, 7]. NVAF is associated with a five times greater risk of ischemic stroke, and NVAF is the main contributing factor for up to 25% of strokes in patients over the age of 80 years [8].

Current treatment options to reduce the occurrence of strokes related to NVAF include medical and surgical therapies. The use of anticoagulation, although effective in preventing stroke, has been associated with bleeding as well as challenges with patient noncompliance (e.g. taking their medications) [9, 10]. Alternatively, a number of mechanical devices have been developed that are designed to prevent thrombus by occluding or excluding the LAA. Current AHA/ACC Guidelines recommend the use of percutaneous LAA occlusion devices for patients who are poor candidates for anticoagulant therapy due to propensity for bleeding or poor drug tolerance and/or adherence [11]. While the procedure is conceptually simple, development of devices that provide effective anatomic fit and placement (device-ostium shape match) and reliably achieve complete occlusion (no leak) is challenging due in part to the variability of LAA shape and ostium dimensions [12, 13].

To overcome these challenges, the *StrokeShield* system is being developed, which is comprised of a LAA closure device that fully occludes the LAA orifice and collapses the LAA chamber, independent of orifice geometry and without obstructing the pulmonary veins or mitral valve, and a transseptal catheter-based delivery tool. *StrokeShield* is designed to completely collapse and seal the LAA, which may help lead to further reductions in the incidence of thromboembolic stroke and adverse events and may potentially enable earlier weaning from anticoagulation. In this article, the basic concept, prototype design, and proof-of-concept testing of the *StrokeShield* system is presented.

## Materials and methods

### Device design

**Fig 1:**
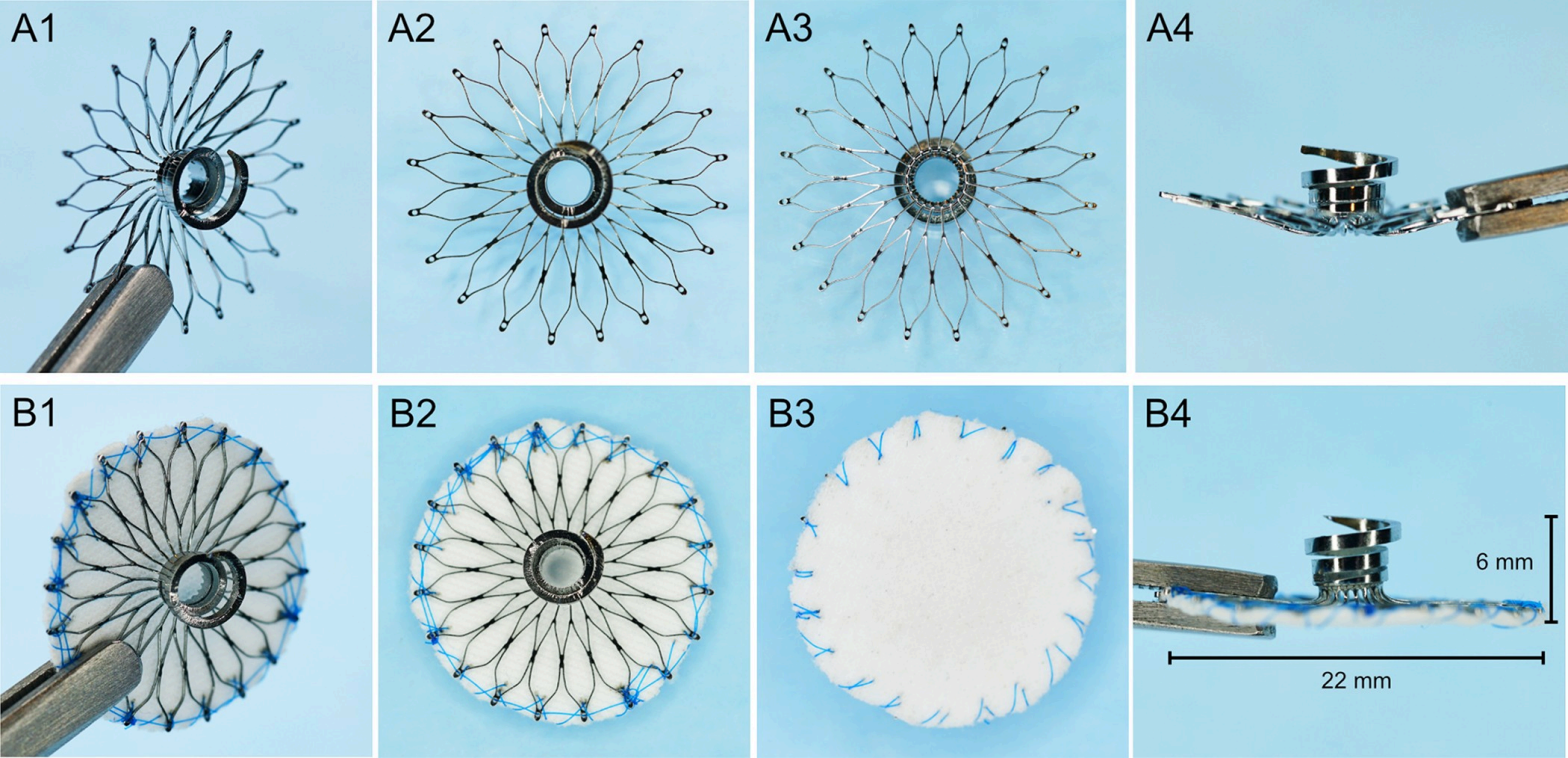
*StrokeShield* device. The two primary components of the StrokeShield device (22 mm width, 6 mm height) include conical anchor which screws into the LAA myocardium to secure the device, and a Dacron-covered Nitinol umbrella membrane that unfolds following deployment to occlude and collapse the LAA. The upper (A1-A4) and lower (B1 = B4) photos show device with bare Nitinol wire and Dacron-covered umbrella frame, respectively, for isometric (A1,B1), front (A2,B2), back (A3,B3), and side (A4,B4) views.

The *StrokeShield* system (US patents 10,898,202, 10,531,878) features of an expanding circular umbrella closure device to occlude the LAA ostium and a coil anchor to secure and collapse the LAA wall for closure of the LAA with complete seal (tissue integration) and no residual chamber space (eliminate LAA volume/prevent peri-device leak), Fig 1. The umbrella is constructed from a single Nickel-Titanium (Nitinol) tube with an expanding lattice design to provide structural integrity and is covered with Dacron to facilitate tissue in-growth and encapsulation. The umbrella is designed to completely cover the LAA ostium and will be available in multiple sizes (21, 28, 33mm) with oversize fit (20% larger than LAA orifice) to meet the expected range of patient LAA geometries and sizes (16-36mm) [14, 15]. The super-elastic Nitinol design enables the LAA closure device to be pre-collapsed in the forward axial direction of the delivery catheter, which will enable all of the implant devices (independent of size) to be deployed using the same delivery tool via a steerable 12Fr sheath. A conical coil cut into the opposing end of the Nitinol tube is designed to secure the LAA closure device to the LAA wall. The collapsible LAA closure device (umbrella) and coil anchor are pre-loaded on a delivery tool. The anchor is secured to the LAA free wall by a clinician applying rotational force using the hand-held control knob to embed the coil anchor. The 2.5-turn coil provides 35mm^2^ of anchoring surface area designed to have greater pull-out force than suture in cardiac tissue thereby reducing the risk of device migration or myocardial tear. The coil configuration and geometry are designed to compress the LAA wall by creating an outward tissue dimple on the external surface of the LAA wall due to radial myocardial compression. The anchoring coil design is based upon the successful history of other proven implantable medical device technologies that also use screw-in coil mechanisms (i.e. pacing leads [16], apical closure devices [17]) with an established track record of minimal leaks, superior strength, and the ability to be easily retrievable. Only a single contact point is required to secure the Dacron-covered umbrella to the LAA wall, occlude the ostium, and collapse the LAA wall. This approach is designed to reduce the risk of bleeding or tamponade, but will require extensive pre-clinical testing to demonstrate efficacy and safety.

This study was carried out in accordance with the recommendations in the Guide for the Care and Use of Laboratory Animals of the National Institutes of Health. The protocol was approved by the Institutional Animal Care and Use Committee at the AAALAC-accredited University of Louisville (OLAW/PHS Assurance No. A3586-01).

#### Acute *in-vivo* testing

Two acute non-survival experiments were performed in a healthy canine model to demonstrate intraoperative proof-of-concept of the *StrokeShield* LAA closure device. Two healthy male dogs (34kg, mongrel hounds, Oak Hill Genetics, Ewing IL) underwent ketamine-diazepam sedation followed by endotracheal intubation for mechanical ventilation under general isoflurane anesthesia. Introducers were placed in the carotid artery and jugular vein for drug/fluid administration, blood draws, and cardiac access. A right thoracotomy was performed, and a purse string suture was placed in the LA dome. Heparin was administered and a needle and wire were inserted into the LA dome. The entry point was serially dilated to accommodate the deployment sheath. The device was placed and deployed into the LAA ostium with secure positioning documented by transesophageal echocardiography (TEE) and fluoroscopy. The device remained *in situ* for a minimum of four hours intraoperatively, after which the animals were euthanized under general anesthesia (IV Beauthanasia D-special, 1mL/10lbs) and necropsies were performed. At necropsy, a device pull-out test was performed.

#### Chronic *in-vivo* testing

Two 60-day chronic survival experiments were performed in a healthy canine model to evaluate long-term proof-of-concept of the *StrokeShield* LAA closure device. Two healthy male dogs (27kg, mongrel hounds, Oak Hill Genetics, Ewing IL) underwent LAA device placement as described above. Following device placement, the animals’ thoracotomy incisions were closed, a thoracic catheter was placed for the first post-operative day, the neck lines were removed, post-operative analgesia (fentanyl patch) was initiated, and the animals were extubated and recovered from anesthesia. The animals were maintained post-operatively as needed on aspirin (81mg oral to maintain platelet count within 200–500 K/μL and/or in response to oozing or bleeding at the incision site) and/or Coumadin (2mg oral to maintain INR (international normalized ratio) target range of 2–3). Sixty days later, the animals returned to the fluoroscopy suite and were anesthetized as described above for terminal TEE and fluoroscopic imaging. Following image acquisition, the animals were euthanized under general isoflurane anesthesia (IV Beauthanasia D-special, 1mL/10lbs) and necropsies were performed. Tissue samples were collected for Hematoxylin and Eosin (H&E) and elastic trichrome staining.

## Results and discussion

### Results

#### Acute *in-vivo* testing

Intraoperative placement of the *StrokeShield* device via the LA dome approach was demonstrated. Acute results demonstrated that the conical coil securely attached to the myocardium and the Nitinol umbrella fully deployed and covered the LAA ostium. At necropsy, up to 5N (510 gram) of pull-out force was applied to the device without tearing LAA tissue or extracting the device demonstrating secure attachment of the coil in the LAA myocardium.

#### Chronic *in-vivo* testing

**Fig 2:**
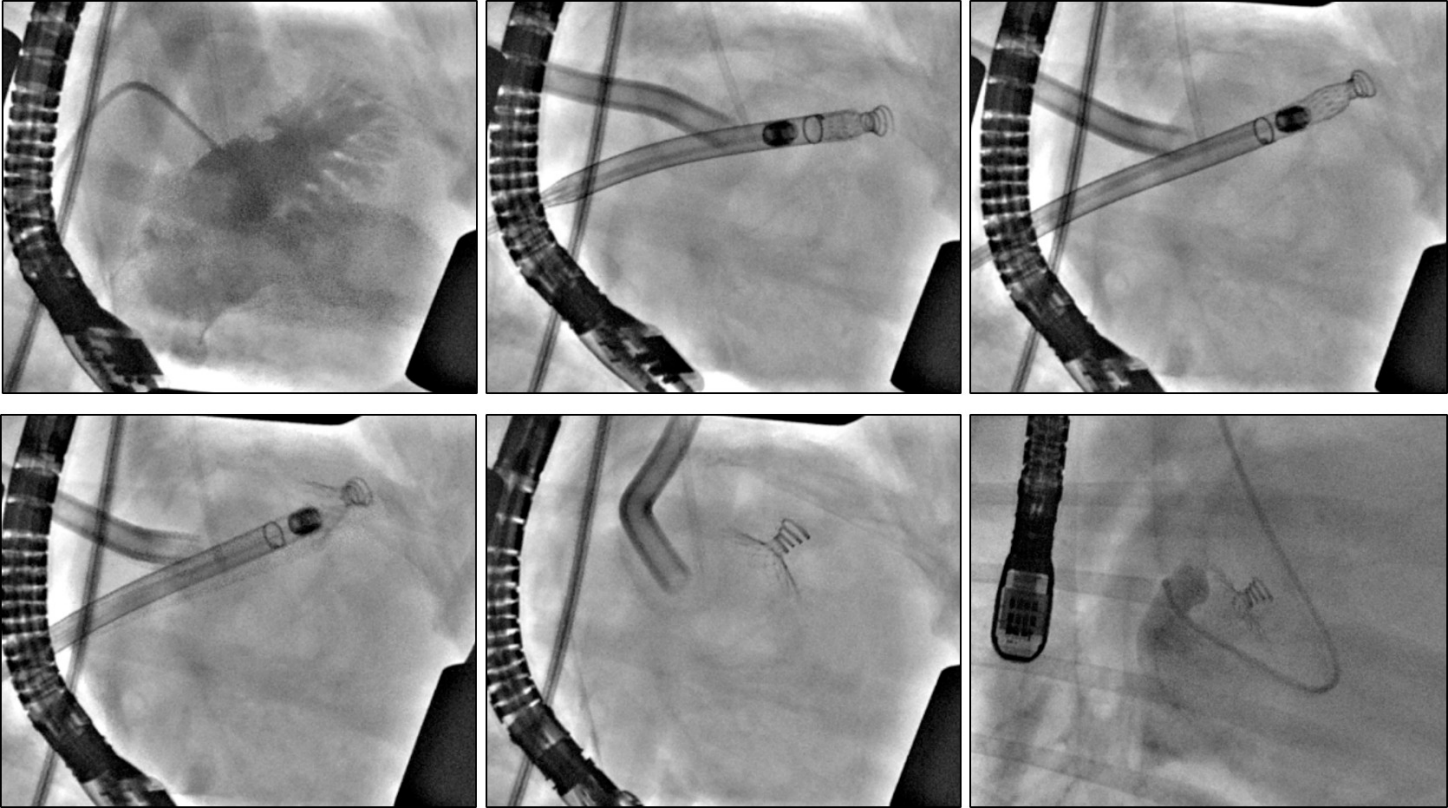
*In vivo* deployment of the *StrokeShield* device. Fluoroscopic images from 60-day chronic animal study showing *in vivo* deployment of the *StrokeShield* device. Panels A-E were obtained at baseline/implant and panel F at day-60 (terminal study). A, contrast dye injection into the LA showing the LAA. B, Direct surgical approach via the LA dome for placement of the *StrokeShield* device. The device is loaded on the tip of the delivery tool with partial exposure of the conical coil. C, *StrokeShield* device screwed into the LAA myocardium with delivery tool pullback in progress. D. Partial opening of the *StrokeShield* Nitinol umbrella. E. Complete opening of the *StrokeShield* Nitinol umbrella in place in the LAA ostium. F, *StrokeShield* device at day-60. Contrast dye injected via a ventriculogram catheter into the LA chamber shows no dye flow into the collapsed LAA. Panel F, originally obtained in right lateral decubitus position, has been flipped horizontally to match the left lateral decubitus position (right thoracotomy) of panels A-E.

**Fig 3:**
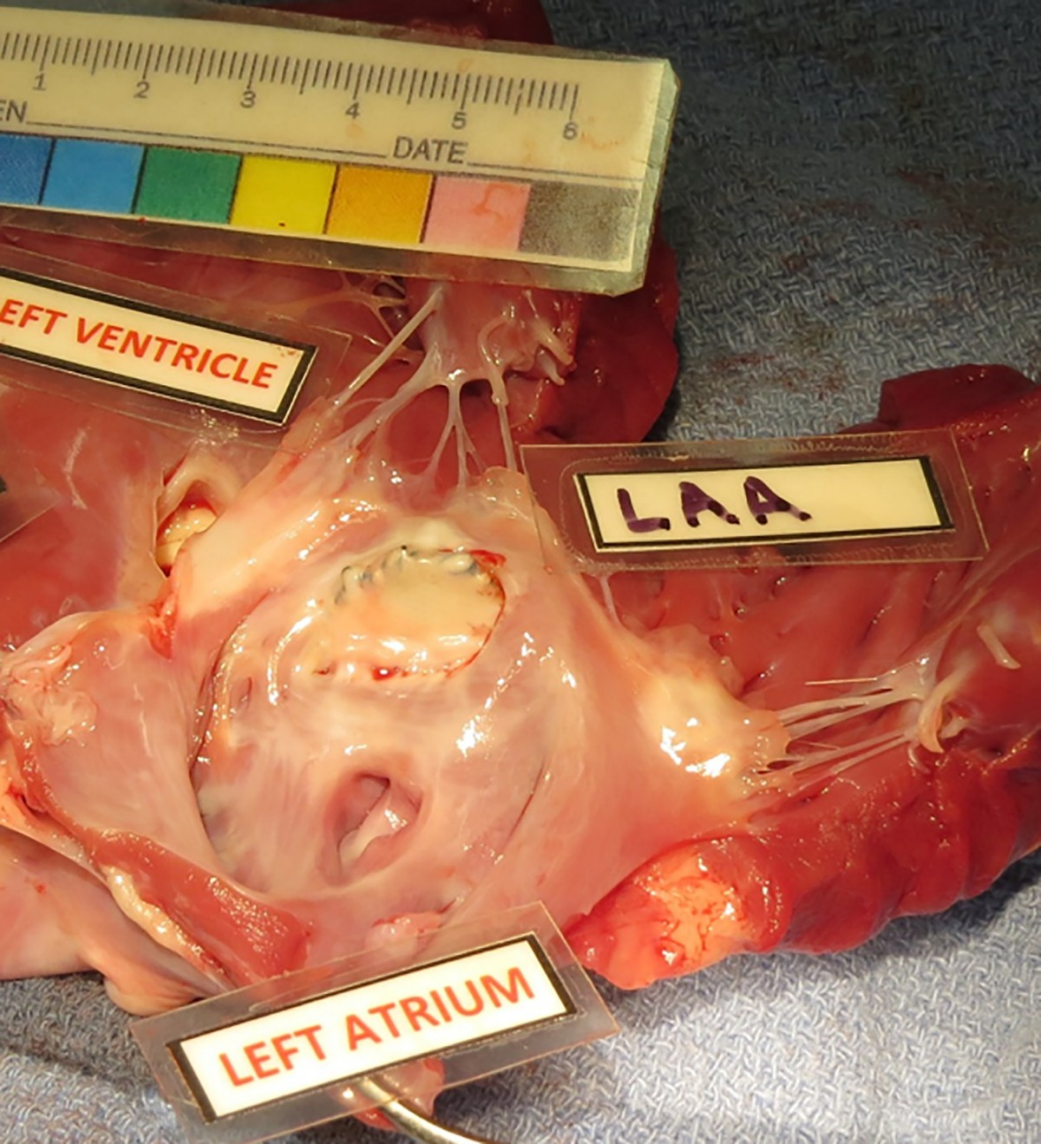
*StrokeShield* device *in situ*. The *StrokeShield* device in the LAA at necropsy (day 60) in the chronic canine model demonstrating tissue encapsulation and freedom from interference with the mitral valve and pulmonary veins.

**Fig 4:**
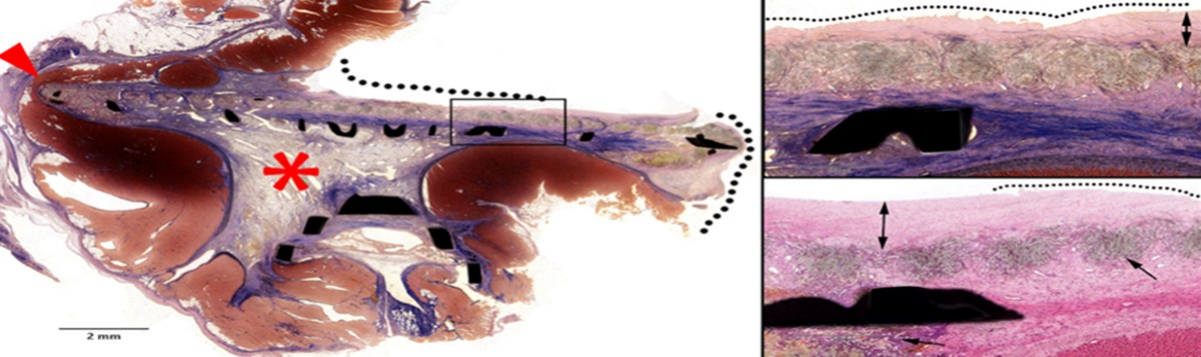
Histology performed on 60-day chronic canine myocardium and *StrokeShield* device. At left, Elastic Trichrome (ET) stain demonstrating healed and mature neointima covering the Dacron and nitinol struts (dark black shapes) of the closure device (black dotted line), fully mature fibrovascular connective tissue filling the closure device cavity (red asterisk), and occlusion point at the contact between closure device disc and atrial wall with normal healing (red arrowhead). The black box is further detailed in the upper right image (ET stain). Here, the dotted line indicates healed and mature neointima covering the closure device with irregular edges and microtears indicative of handling artifact. The black double arrow is mature fibrocellular neointima lining the Dacron cover. At lower right, Hematoxylin and eosin stain (H&E) (lower right) showing healed and mature neointima (dotted line) covering the closure device with irregular edges and microtears indicative of handling artifact. The black double arrow shows mature fibrocellular neointima lining the Dacron cover and the single arrow shows Dacron with expected minimal to mild foreign body inflammatory response [18].

Long-term feasibility of device placement with no procedural or device-related intra- or post-operative complications was demonstrated. The animals demonstrated normal cardiac and neurological exams across the 60-day study. Both TEE and fluoroscopy (Fig 2) demonstrated secure placement and correct positioning of the device with no device migration or peri-device flow. The device successfully occluded the LAA ostium and collapsed the LAA with no interference with the mitral valve, circumflex coronary artery, or pulmonary veins (Fig 3). There was no evidence of device/coil perforation or gross LAA cardiac tissue injury or pericardial effusion at implant, during the chronic 60d test period, or at terminal necropsy. Necropsy demonstrated no gross signs of thrombus or end-organ damage. The device was encapsulated in the LAA. Histology using Hematoxylin and Eosin stain (H&E) and Elastic Trichrome stain was performed. Findings demonstrated healed and mature neointima covering the Dacron and Nitinol struts of the closure device. Mature fibrovascular connective tissue filled the closure device cavity and occlusion point at the contact between closure device disc and atrial wall with normal healing. Mature neointima covered the closure device and mature fibrocellular neointima lined the Dacron cover with expected minimal to mild foreign body response (Fig 4) [18].

## Discussion

Between 2004–10, the estimated prevalence of atrial fibrillation (AF) in the US was 4.3 million with nearly 700,000 undiagnosed cases (13%), and its occurance increases with age [19]. In their review of the epidemiology of AF, Kronej et al. report that as survival with chronic diseases in the elderly population has improved the incidence and prevalence of AF continues to increase, prompting them to suggest AF is becoming a “global epidemic” [20]. Currently, the commercial landscape in the NVAF field of mechanical device therapies, including Watchman™, Amplatzer™, Occlutech™, WaveCrest™, LAmbre™, and Ultraseal™ devices, indicating a robust interest by clinicians and their patients in the continued development and propagation of LAA mechanical device technologies. The Watchman is the only FDA approved LAA closure device with PROTECT [21] and PREVAIL [22] randomized clinical trials demonstrating non-inferiority to oral anticoagulant (warfarin) for ischemic stroke protection but not overall efficacy. Recently, a meta-analysis comparing Watchman and Amplatzer devices for stroke protection resulted in similar low complication and event rates findings with comparable efficacy and safety results [23]. There are also many other emerging LAA mechanical device technologies in various stages of pre-clinical development [24–28].

LAA occlusion devices are designed to block and/or fill the LAA ostium, which if not completely occluded, can result in blood leakage and stagnation near the exposed surrounding edges of the LAA orifice increasing the potential risk for thrombogenesis (and stroke). LAA devices with membrane-covered frames may only partially fill the LAA chamber (leaving residual volume) with potential risk for developing a large thrombus within the LAA cavity following occlusion. The LAA tissue, geometric shape, and size of the LAA ostium varies from patient-to-patient making accurate sizing and fixation of the implant challenging as well as risk of the device perforating and/or causing tissue injury. The incidence of pericardial effusion requiring surgery, need for pericardiocentesis, and device embolization have been reported to be 0.4–1.6%, 1.5–2.9%, and 0.4–0.7%, respectively, in clinical trials [29]. Despite the clinical limitations and technological challenges of LAA mechanical devices, there is strong clinical interest and justification for their continued development and advances field [30].

The *StrokeShield* system combines the advantages of LAA exclusion (surgical) and occlusion (catheter-based delivery) devices into a single LAA closure procedure by completely collapsing the LAA between the ostium (umbrella blocks opening) and free wall (conical coil) with a secure anchoring mechanism. The unicity of the StrokeShield system is its hybrid concept (LAA collapse and occlusion), advancement of technology (patent-protected anchor and umbrella design and methods), and potential clinical benefits, including eliminating need for anti-coagulation post-implant). The *StrokeShield* device deploys a conical coil that anchors the device to the LAA wall. The axial length of the conical coil provides up to 35 mm^2^ cross-sectional area within the LA wall to achieve greater than 5N pull-out load while providing a strong and secure single-point attachment to the LAA free wall to reduce the risk of device migration. The axial length of the coil is designed to control depth of penetration into LAA wall (number of coils, conical shape) and provide LAA tissue compression to reduce the risk of pericardial effusion and cardiac tamponade. The *StrokeShield* collapsible occluder device is projected to be fabricated in different patient-specific sizes (21, 28, 33 mm) and implanted in sizes ~20% larger than the LAA orifice and curved toward the LA wall to completely cover the LAA orifice regardless of orifice geometry without obstructing the pulmonary veins or mitral valve. Next design iteration(s) of the collapsible occluder device will be developed for delivery using a steerable, multi-stage catheter delivery tool (12Fr) through femoral vein access. The catheter delivery tool will be designed for advancement through the venous vasculature into the right atrium (RA), curved using a steerable component to allow for transeptal access into the LA, and then used to anchor and deploy the collapsible occluder to completely cover and occlude the LAA ostium and collapse the LAA to eliminate chamber volume and flow.

To demonstrate proof-of-concept, prototype *StrokeShield* devices were tested in healthy acute and 60-day canine models. In the acute experiments, surgical approach with access for delivery and deployment in the LAA was achieved demonstrating proper anatomic fit for closure and exclusion of the LAA ostium without tearing tissue or causing cardiac tamponade. In the chronic experiments, preliminary evaluation of device efficacy and biocompatibility was achieved as evidenced by elimination of LAA residual volume and peri-device flow, and no device migration, perforation, or inflammation. Several challenges associated with prototype devices were identified and engineering solutions for the next series of design iterations are being carefully considered. Specifically, the methodology for pre-loading device into the catheter sheath, proper sizing for a wide range in LAA size and variability in LAA shape, promotion of healthy device encapsulation and endothelialization, reducing the risk for coil perforation, tissue damage, and/or inflammation, and development of a multi-stage delivery tool that enables steering, device retrieval, re-positioning and re-deployment, and prevents over-torquing during device implant are warranted.

## Conclusion

Proof-of-concept of the *StrokeShield* system was demonstrated in acute and chronic animal experiments as evidenced by successful implant procedure and proper anatomic fit with no device migration or perforation, and complete closure and occlusion of the LAA ostium with tissue encapsulation and absence of residual volume and peri-device leak. The continued development of the *StrokeShield* system may lead to an alternative catheter-based approach for percutaneous delivery of a novel LAA device (Dacron covered Nitinol umbrella) that is secured to the LAA wall via a single anchoring point (Nitinol conical coil) to mitigate the risk of stroke originating from the LAA in patients with NVAF.

## References

[1] Suradi HS, Hijazi ZM. Left atrial appendage closure: outcomes and challenges. Neth Heart J. 2016;25: 143–151. doi: 10.1007/s12471-016-0929-0 27943175 PMC5260621

[2] Reddy VY, Akehurst RL, Gavaghan MB, Amorosi SL, Holmes DR Jr. Cost-effectiveness of left atrial appendage closure for stroke reduction in atrial fibrillation: analysis of pooled, 5-year, long-term data. Journal Am Heart Assoc. 2019;8: e011577. doi: 10.1161/JAHA.118.011577 31230500 PMC6662368

[3] Amin A, Keshishian A, Trocio J, Dina O, Le H, Rosenblatt L, et al. Risk of stroke/systemic embolism, major bleeding and associated costs in non-valvular atrial fibrillation patients who initiated apixaban, dabigatran or rivaroxaban compared with warfarin in the United States Medicare population. Curr Med Res Opin. 2017;33: 1595–1604. doi: 10.1080/03007995.2017.1345729 28635338

[4] Wang F, Zhu M, Wang X, Zhang W, Su Y, Lu Y, et al. Predictive value of left atrial appendage lobes on left atrial thrombus or spontaneous echo contrast in patients with non-valvular atrial fibrillation. BMC Cardiovasc Disord. 2018;18: 153. doi: 10.1186/s12872-018-0889-y 30064363 PMC6069846

[5] Gilligan AM, Gandhi P, Song X, Wang C, Henriques C, Sander S, et al. All-cause, stroke-, and bleed-specific healthcare costs: comparison among patients with non-valvular atrial fibrillation (NVAF) newly treated with Dabigatran or Warfarin. Am J Cardiovasc Drugs. 2017;17: 481–492. doi: 10.1007/s40256-017-0244-1 28795348 PMC5701952

[6] January CT, Wann LS, Calkins H, Chen LY, Cigarroa JE, Cleveland JC Jr, et al. 2019 AHA/ACC/HRS focused update of the 2014 AHA/ACC/HRS Guideline for the Management of Patients with Atrial Fibrillation: A Report of the American College of Cardiology/American Heart Association Task Force on Clinical Practice Guidelines and the Heart Rhythm Society in collaboration with the Society of Thoracic Surgeons. Circulation. 2019;140: e125–e151. doi: 10.1161/CIR.0000000000000665 30686041

[7] Willey V, Franchino-Elder J, Fu A-C, Wang C, Sander S, Tan H, et al. Treatment and persistence with oral anticoagulants among newly diagnosed patients with non-valvular atrial fibrillation: a retrospective observational study in a US commercially insured and Medicare Advantage population. BMJ Open. 2018; 8: e020676. doi: 10.1136/bmjopen-2017-020676 29961012 PMC6042605

[8] Czuprynska J, Patel JP, Arya R. Current challenges and future prospects in oral anticoagulant therapy. Br J Haematol. 2017;178: 838–851. doi: 10.1111/bjh.14714 28573648

[9] Chow DHF, Wong Y-H, Park J-W, Lam Y-Y, De Potter T, Rodés-Cabau J, et al. An overview of current and emerging devices for percutaneous left atrial appendage closure. Trends in Cardiovasc Med. 2019;29: 228–236. doi: 10.1016/j.tcm.2018.08.008 30205924

[10] Asmarats L, Rodés-Cabau J. The spectrum of devices for percutaneous left atrial appendage occlusion. Cardiac Interventions Today. 2018;12:34–39.

[11] Reddy VY, Akehurst RL, Armstrong SO, Amorosi SL, Beard SM, Holmes DR Jr. Time to cost-effectiveness following stroke reduction strategies in AF: Warfarin versus NOACs versus LAA closure. J Am Coll Cardiol 2015;66: 2728–39. doi: 10.1016/j.jacc.2015.09.084 26616031

[12] Reynolds SL, Ghate SR, Sheer R, Gandhi PK, Moretz C, Wang C, et al. Healthcare utilization and costs for patients initiating Dabigatran or Warfarin. Health Qual Life Outcomes. 2017;15: 128: doi: 10.1186/s12955-017-0705-x 28637460 PMC5480105

[13] McBride DA, Markman TM, Liang JJ, Santangeli P. Left atrial appendage closure devices for stroke prevention in patients with non-valvular AF. US Cardiology Review. 2018;12: 87–90. doi: 10.15420/usc.2018.6.1

[14] Karim N, Ho SY, Nicol E, Li W, Zemrak F, Markides V, et al. The left atrial appendage in humans: structure, physiology, and pathogenesis. Europace. 2020;22: 5–18. doi: 10.1093/europace/euz212 31578542

[15] Kamohara K, Fukamachi K, Ootaki Y, Akiyama M, Cingoz F, Ootaki C, et al. Evaluation of a novel device for left atrial appendage exclusion: the second-generation atrial exclusion device. J Thorac Cardiovasc Surg. 2006;132:340–6. doi: 10.1016/j.jtcvs.2006.04.021 16872960

[16] Hsia T-Y, Bradley SM, LaPage MJ, Whelan S, Saul JP, Ringewald JM, et al. Novel minimally invasive, intrapericardial implantable cardioverter defibrillator coil system: a useful approach to arrhythmia therapy in children. Ann Thorac Surg. 2009;87: 1234–1238. doi: 10.1016/j.athoracsur.2009.01.015 19324158

[17] Blumenstein J, Kempfert J, Linden AV, Arsalan M, Schmidt SK, Mollmann H, et al. First-in-man evaluation of the transapical APICA ASC^TM^ access and closure device: the initial 10 patients. Eur J Cardiothorac Surg. 2013;44: 1057–1062. doi: 10.1093/ejcts/ezt198 23562938

[18] Klopfleisch R, Jung F. The pathology of the foreign body reaction against biomaterials. J Biomed Mater Res A. 2017;105: 927–40. doi: 10.1002/jbm.a.35958 27813288

[19] Turakhia MP, Shafrin J, Bognar K, Trocio J, Abdulsattar Y, Wiederkehr D, et al. (2018) Estimated prevalence of undiagnosed atrial fibrillation in the United States. PLoS ONE 13(4): e0195088. doi: 10.1371/journal.pone.0195088 29649277 PMC5896911

[20] Kornej J, Borschel CS, Benjamin EJ, Schnabel RB. Epidemiology of atrial fibrillation in the 21^st^ century: novel methods and new insights. Circulation Research. 2020; 127: 4–20. doi: 10.1161/CIRCRESAHA.120.316340 32716709 PMC7577553

[21] Romero J, Perez IE, Krumerman A, Garcia MJ, Lucariello RJ. Left atrial appendage closure devices. *Clin Med Insights Cardiol*. 2014;8: 45–52. doi: 10.4137/CMC.S14043 24963274 PMC4064949

[22] Holmes DR, Reddy VY, Turi ZG, Doshi SK, Sievert H, Buchbinder M, et al. Percutaneous closure of the left atrial appendage versus warfarin therapy for prevention of stroke in patients with atrial fibrillation: a randomized non-inferiority trial. *Lancet*. 2009;374: 534–42. doi: 10.1016/S0140-6736(09)61343-X 19683639

[23] Basu Ray I, Khanra D, Shah S, Char S, Jia X, Lam W, et al. Meta-Analysis Comparing Watchman^TM^ and Amplatzer Devices for Stroke Prevention in Atrial Fibrillation. Front Cardiovasc Med. 2020 Jun 22;7:89. doi: 10.3389/fcvm.2020.00089 ; PMCID: PMC7322993 32656246 PMC7322993

[24] Asmarats L, Rodes-Cabau J. The spectrum of devices for percutaneous left atrial appendage occlusion. *Cardiac Interventions Today*. 2018; 12(3): 34–39.

[25] Chow D, Wong YH, Park JW, Lam YY, DePotter T, Rodes-Cabau J, et al. An overview of current and emerging devices for percutaneous left atrial appendage closure. *Trends in Cardiovascular Medicine*. 2019; 29: 228–36. doi: 10.1016/j.tcm.2018.08.008 30205924

[26] Karim N, Ho SY, Nicol E, Li W, Zemrak F, Markides V, et al. The left atrial appendage in humans: structure, physiology, and pathogenesis. Europace. 2019; 0: 1–14. doi: 10.1093/eurospace/euz212 31578542

[27] Sharma SP, Park P, Lakkireddy D. Left atrial appendages occlusion: current status and propspective. *Korean Circulation Journal*. 2018;48(8): 692–704. doi: 10.4070/kcj.2018.0231 30073807 PMC6072669

[28] Tan NY, Yasin OZ, Sugrue A, El Sabbagh A, Foley TA, Asirvatham SJ. Anatomy and physiologic roles of the left atrial appendage: Implications for endocardial and epicardial device closure. *Intervent*. *Cardiol*. *Clin*. 2018;7: 185–99. doi: 10.1016/j.iccl.2017.12.001 29526287

[29] Holmes DR, Kar S, Price MJ, Whisenant B, Sievert H, Doshi SK, et al. Prospective randomized evaluation of the Watchman Left Atrial Appendage Closure device in patients with atrial fibrillation versus long-term warfarin therapy: the PREVAIL trial. *J Am Coll Cardiol*. 2014;64(1): 1–12. doi: 10.1016/j.jacc.2014.04.029 24998121

[30] Atianzar K, Fafoor S. Why do we need yet another left atrial appendage occluder device? *JACC*: *Cardiovascular Interventions*. 2018;11(19): 1942–44. doi: 10.1016/j.jcin.2018.07.005 30249440

